# Infection with Usutu Virus Induces an Autophagic Response in Mammalian Cells

**DOI:** 10.1371/journal.pntd.0002509

**Published:** 2013-10-24

**Authors:** Ana-Belén Blázquez, Estela Escribano-Romero, Teresa Merino-Ramos, Juan-Carlos Saiz, Miguel A. Martín-Acebes

**Affiliations:** Departamento de Biotecnología, Instituto Nacional de Investigación y Tecnología Agraria y Alimentaria (INIA), Madrid, Spain; University of Texas Medical Branch, United States of America

## Abstract

Usutu virus (USUV) is an African mosquito-borne flavivirus closely related to West Nile virus and Japanese encephalitis virus, which host range includes mainly mosquitoes and birds, although infections in humans have been also documented, thus warning about USUV as a potential health threat. Circulation of USUV in Africa was documented more than 50 years ago, but it was not until the last decade that it emerged in Europe causing episodes of avian mortality and some human severe cases. Since autophagy is a cellular pathway that can play important roles on different aspects of viral infections and pathogenesis, the possible implication of this pathway in USUV infection has been examined using Vero cells and two viral strains of different origin. USUV infection induced the unfolded protein response, revealed by the splicing of Xbp-1 mRNA. Infection with USUV also stimulated the autophagic process, which was demonstrated by an increase in the cytoplasmic aggregation of microtubule-associated protein 1 light chain 3 (LC3), a marker of autophagosome formation. In addition to this, an increase in the lipidated form of LC3, that is associated with autophagosome formation, was noticed following infection. Pharmacological modulation of the autophagic pathway with the inductor of autophagy rapamycin resulted in an increase in virus yield. On the other hand, treatment with 3-methyladenine or wortmannin, two distinct inhibitors of phosphatidylinositol 3-kinases involved in autophagy, resulted in a decrease in virus yield. These results indicate that USUV virus infection upregulates the cellular autophagic pathway and that drugs that target this pathway can modulate the infection of this virus, thus identifying a potential druggable pathway in USUV-infection.

## Author Summary

The identification of cellular components and metabolic pathways involved in virus replication provides valuable information for the development of new antiviral strategies. Autophagy is one of these metabolic pathways with multiple implications during viral replication. Autophagy literally means self-digestion and constitutes a cellular process by which intracellular components are enclosed by membrane structures and degraded. Interestingly autophagy can contribute either positively or negatively to viral infections. For instance, several viruses hijack these autophagic membranes to build their replication complexes or take advantage on metabolic rearrangements induced following autophagy, while in other cases autophagy contributes to viral clearance and innate immunity. In this study, we explored the possible implication of the autophagic pathway during Usutu virus infection (USUV). USUV is an African mosquito-borne flavivirus that mainly infects mosquitoes and birds, although infections in humans have been also documented, thus warning about USUV as a potential health threat. Our results indicate that infection by USUV of different origins triggers an autophagic response within infected cells. Even more, drugs that target components from the autophagic pathway modulate USUV-infection. These results provide the basis for the design of new antiviral research lines against this pathogen.

## Introduction

The variety of factors that have contributed to the emergence of the flavivirus West Nile virus (WNV) in the Americas and its re-emergence in other parts of the world could also provide a suitable scenario for the emergence of other arboviruses [1], [2], [3]. These potential threats for human and animal health include other related mosquito-borne viruses such as Usutu virus (USUV) [4]. USUV is an enveloped single-stranded positive polarity RNA virus that belongs to the *Flavivirus* genus in the *Flaviviridae* family. USUV was first described in South Africa in 1959, and since then, it has been reported in several African countries including Senegal, Central African Republic, Nigeria, Uganda, Burkina Faso, Cote d'Ivore, and Morocco [5]. The host range of USUV in Africa mainly comprises ornitophilic *Culex* mosquitoes and birds, although two isolations of USUV from human serum, including one severe case, have been documented [5]. USUV was reported to be circulating only in Africa until 2001, when it emerged in Central Europe [6]. From that time-point, USUV has been detected in several European countries often associated to episodes of avian mortality [4], [6]. There is also increasing evidence of virus circulation among horses and humans in Europe [7], [8], [9], [10] and recently two cases of neuroinvasive disease in humans have been documented [11], [12]. This current scenario reinforces the notion that USUV can infect humans and play a role as a pathogen capable to induce a broad spectrum of symptoms that range from fever, rash or jaundice to meningoencephalitis [5], [11], [12]. Albeit the number of cases of human USUV infections is rather limited, the similarities of USUV ecology with that of WNV emphasize the need to be cautious about its potential threat to human health [4], [5]. Even more, the observed symptoms of human USUV infections are not very specific, which could have probably led to an underestimation of the infections in endemic areas, which mainly comprise developing countries.

A detailed knowledge of the cellular processes involved in pathogen and host cell interactions is desirable to design effective strategies to combat arboviral diseases. In the case of USUV, the role of many aspects of the interaction between the virus and the host cell, for instance its relationship with the autophagic pathway, remains to be explored. Macroautophagy (thereafter referred as ‘autophagy’) is a cellular process by which cytoplasmic components are sequestered into double-membrane vesicles and degraded to maintain cellular homeostasis. In addition to this, autophagy constitutes an evolutionarily ancient process for survival during different forms of cellular stress, including infection with viruses [13], [14]. As a first line of defence against intracellular pathogens, autophagy can contribute to viral clearance through the degradation of viral components located in the cell cytoplasm [13]. But antiviral aspects of autophagy go beyond, and this catabolic route has been also implicated in both innate and adaptive immunity, i.e. by promoting the delivery of Toll-like receptor (TLR) ligands to endosomes, or by feeding antigens to MHC class II pathway [13], [15], [16]. Autophagy can also extend the survival of infected cells by limitation of apoptosis [17]. Conversely, some viruses can take advantage on the induction of autophagy by co-opting components from the autophagic machinery in their own benefit to provide the adequate cellular platforms for replication [18], [19], [20] or by rearrangement of cellular lipid metabolism in order to support strong viral replication [21]. All these features make of autophagy a relevant druggable metabolic pathway during multiple human disorders, including viral infections, so interventions on this route could constitute potential therapies [14], [16], [22].

Regarding the *Flaviviridae*, autophagy has been associated to different aspects of the replication and pathogenicity of some members of this virus family, including Dengue virus (DENV) [21], [23], [24], [25], Modoc virus [24], Japanese encephalitis virus (JEV) [26], and hepatitis C virus (HCV) [27], [28]. In the case of WNV, the induction or not of an autophagy response remains contentious. One recent report pointed that WNV infection induced an autophagic response [29], whereas another suggested that the autophagic pathway was not upregulated in WNV-infected cells [30]. Relative to USUV, to our knowledge, the involvement of the autophagic machinery during its replication has not been previously documented.

In this study we have analyzed the induction of autophagy following infection with a prototypic African strain of USUV and a recent European isolate [31]. The ability of both strains to provoke an autophagic response on infected cells was documented. Even more, pharmacological intervention at the autophagic pathway modulated USUV infection, thus identifying a cellular pathway for potential interventions on USUV infection.

## Methods

### Ethics statement

All animals were handled in strict accordance with the guidelines of the European Community 86/609/CEE at the biosafety animal facilities of the Centro de Investigación en Sanidad Animal of the Instituto Nacional de Investigación Agraria y Alimentaria (CISA-INIA). The protocols were approved by the Committee on Ethics of Animal Experimentation of INIA (permit number 2013–015).

### Antibodies

Mouse monoclonal antibody J2 against double-stranded RNA (dsRNA) was purchased from English & Scientific Consulting (Hungary). Rabbit monoclonal anti-LC3B, rabbit anti-p62/SQSTM1 and mouse monoclonal anti-β-actin antibodies were from Sigma-Aldrich (St. Louis, MO). Rabbit anti-calnexin antibody was from ECM Biosciences (Versailles, KY). Polyclonal serum from a mice experimentally infected with USUV SAAR 1776 (INIA permit number 2013–015) was also used to detect USUV proteins. Secondary antibodies against Mouse or Rabbit IgGs coupled to Alexa Fluor-488, -594 or -647 were purchased from Life Technologies (Molecular Probes, Eugene, O). Anti-rabbit and anti-mouse secondary antibodies coupled to horseradish peroxidase were from Dako and Sigma, respectively.

### Cells, viruses and infections

All manipulations of infectious virus were carried out in Biosafety level 3 (BSL-3) containment facilities. USUV strain SAAR 1776 (GenBank acc: AY453412.1), the reference South African strain of USUV, and the Austrian strain of USUV Vienna 2001-blackbird (USUV 939/01, GenBank acc: AY453411.1), a recent European isolate of USUV [31] were propagated five and three times, respectively, in Vero cells [32]. Viruses were used at a multiplicity of infection (MOI) of 5 PFU/cell in microscopy experiments and of 0.5 PFU/cell in the rest of experiments. Infections and virus titrations on semisolid agar medium were performed as previously described [33]. Cells were routinely tested for mycoplasma with Mycoalert Mycoplasma Detection Kit (Lonza, Rockland, ME).

### Drug treatments

Autophagy inhibitors 3-methyladenine (3-MA) and wortmannin, and autophagy inducer rapamycin, were purchased from Sigma and used at the concentrations of 2.5 mM, 0.5 µm and 100 ng/ml, respectively. Amonium chloride (NH_4_Cl, Merck) was used at 25 mM. Cells were infected, or mock-infected, and drugs were added to the medium after the first hour of infection. Stock solutions of wortmannin and rapamycin were prepared in dimethyl sulfoxide (DMSO), and DMSO was also used as control in non-treated cells (drug vehicle). Tunicamycin (Sigma), an inducer of unfolded protein response, was also dissolved in DMSO and used at 10 µg/ml. The viability of cells with or without treatment was tested with CellTiter-Glo Luminiscent Cell Viability Assay (Promega).

### Plasmids and transfections

A plasmid encoding GFP-LC3 [34] was transfected to visualize autophagosome formation. Plasmid encoding mCherry-GFP-LC3 was used to detect acidified autophagosomal structures [35]. Fugene HD (Promega, Madison, WI) was used as transfection reagent according to the instructions provided by the manufacturer. Cells were infected or treated with the drugs 24 h post-transfection.

### Immunofluorescence

Assays were carried out as described [32]. Briefly, cells grown on glass cover slips were washed with PBS and fixed with 4% paraformaldehyde in PBS for 15 min at room temperature. Fixed cells were washed with PBS and permeabilized with BPTG (1% BSA, 0.1% TritonX-100, 1M glycine in PBS) for 15 min. Cells were incubated with primary antibody diluted in 1% BSA in PBS for 1 hour. After washing, cells were incubated with fluorescently conjugated secondary antibody for 45 min at room temperature. Samples were mounted with Fluoromount-G (SouthernBiotech, Birmingham, AL) and observed using a Leica TCS SPE confocal laser-scanning microscope. Images were acquired using Leica Advanced Fluorescence Software. Images were processed using ImageJ (http://rsbweb.nih.gov/ij/) and Adobe Photoshop CS2.

### Transmission electron microscopy

Vero cells infected with USUV (MOI of 5 PFU/cell) were washed and fixed 24 h p.i. (30 min at 37°C) in 4% paraformaldehyde-2% glutaraldehyde in 0.1 M phosphate buffer pH 7.4 plus 5 mM CaCl_2_. Cells were scrapped from the flasks and post-fixed in 1% osmium tetroxide-1% potassium ferricyanide (1 h at 4°C), washed three times with bidistilled water and treated with 0.15% tanic acid (1 min). Cells were washed with the buffer and with bidistilled water and stained with 2% uranyl acetate (1 h). Samples were washed and then dehydrated in ethanol and embedded in the resin. Samples were examined using a Jeol JEM-1010 electron microscope (Jeol, Japan) operated at 80 kV and images were acquired using a digital camera 4K×4K TemCam-F416 (Tietz Video and Image Processing Systems GmbH, Gauting, Germany).

### Western blot

Western blot were performed as reported [32]. Cells were lysed on ice in RIPA buffer (150 mM NaCl, 5 mM β-mercaptoethanol,1% NP-40, 0.1% sodium dodecyl sulfate [SDS], 50 mM Tris-HCl pH 8) supplemented with cOmplete protease inhibitor cocktail tablets (Roche, Indianapolis, IN) and Benzonase Nuclease (Novagen, EMD Chemicals, San Diego, CA). Protein concentration was determined by Bradford assay. Equal amounts of proteins were mixed with Laemmli sample buffer, subjected to SDS-PAGE and electrotransferred onto a nitrocellulose or a PVDF membrane. Membrane was blocked with 5% skimmed milk in PBS 0.05% Tween-20, incubated with primary antibodies (overnight at 4°C), washed three times with PBS-Tween, and subsequently incubated with secondary antibodies coupled to horseradish peroxidase (1 h at RT) diluted in 1% skimmed milk in PBS-Tween. Membrane was washed three times and proteins were detected by chemiluminiscence using a ChemiDocTM XRS+ System (Bio-Rad, Hercules, CA). Intensity of protein bands was quantified using ImageLab software 2.0.1 (Bio-Rad).

### Detection of Xbp-1 mRNA

RNA was extracted from control and infected cells with TriPure Isolation Reagent (Roche, Mannheim, Germany) as indicated by the manufacturer. Reverse transcriptions PCR reactions (RT-PCR) were carried out using the SuperScript One Step RT-PCR (Life Technologies). Unspliced or spliced Xbp-1 mRNA was amplified as described [36]. Amplification of GAPDH mRNA was carried as a control for RNA extraction. PCR products were resolved by electrophoresis in a 2% agarose gel.

### Statistical analyses

Data are presented as mean ± standard error of the mean (SEM). To test the significance of the differences, analysis of the variance (ANOVA) was performed with statistical package SPSS 15 (SPSS Inc, Chicago IL) applying Bonferroni's correction for multiple comparisons. Statistically significant differences were considered at P<0.05.

## Results

### USUV-infected cells exhibit accumulation of cellular organelles compatible with autophagic processes

**Figure 1:**
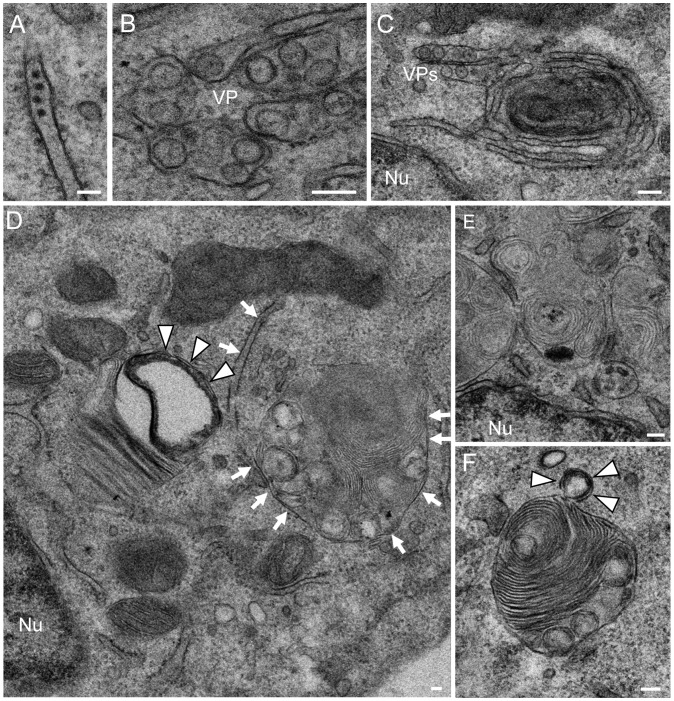
Ultrastructural analysis of USUV-infected cells reveals multiple organelles compatible with an autophagic response. Representative images of Vero cells infected with USUV SAAR 1776 (MOI of 5 PFU/cell) fixed and processed for transmission electron microscopy at 24 h p.i. (A) Electron dense virions located inside endoplasmic reticulum. (B) Vesicle packets (VP). (C) Endoplasmic reticulum-like structure wrapping around cytoplasmic material. Note the presence of VPs associated to the membranes. (D) Cytoplasmic portion showing a double membrane autophagosome-like vacuole (arrowheads) and a double membrane phagophore-like structure engulfing a cytoplasmic portion (arrows). Note also the presence of multi-lamellar structures. (E) Accumulation of multi-lamellar structures. (F) Double membrane vesicle (arrowheads) in association with a multi-lamellar structure. Nu denotes the cell nucleus. Scale bars: 100 µm.

As a first approach, cells infected with USUV strain SAAR 1776 were analyzed by transmission electron microscopy. Infected cells exhibited morphological characteristics associated to flavivirus infection. These included electron dense virions located inside endoplasmic reticulum cisternae (Fig. 1A) and membrane enclosed groups of spherical vesicle-like structures (Fig. 1B). These clusters of vesicles correspond to vesicle packets (VPs) observed for WNV [32], [37], which have been also named double membrane vesicles (DMVs) in the case of DENV [38]. In addition to these classical features associated to flavivirus replication, an accumulation of cellular organelles morphologically related to those associated to degradative processes was patent in USUV-infected cells (Fig. 1C–F). Rearrangement of endoplasmic reticulum-derived structures wrapping around cytoplasmic material was observed (Fig. 1C), providing images typical of autophagic processes. Notably these membranes could be also observed continuous with the membrane of VPs. Membrane rearrangements included double membrane vacuoles (Fig. 1D, arrowheads) compatible with the morphology of autophagosomes. Double membranes engulfing cytoplasmic portions that resemble phagophore-like structures, which constitute the first stages of the formation of autophagosomes, were also observed (Fig. 1D, arrows). In addition to these, multi-lamellar structures constituted by smooth stacked membranes, which have been also associated to autophagic processes [39], were accumulated in the cytoplasm of USUV-infected cells (Fig. 1E), and even observed in association with double membrane vesicles (Fig. 1F, arrowheads). Taken together, the ultrastructural alterations observed in USUV-infected cells are compatible with the activation of an autophagic response.

### Infection with USUV induces LC3 accumulation

**Figure 2:**
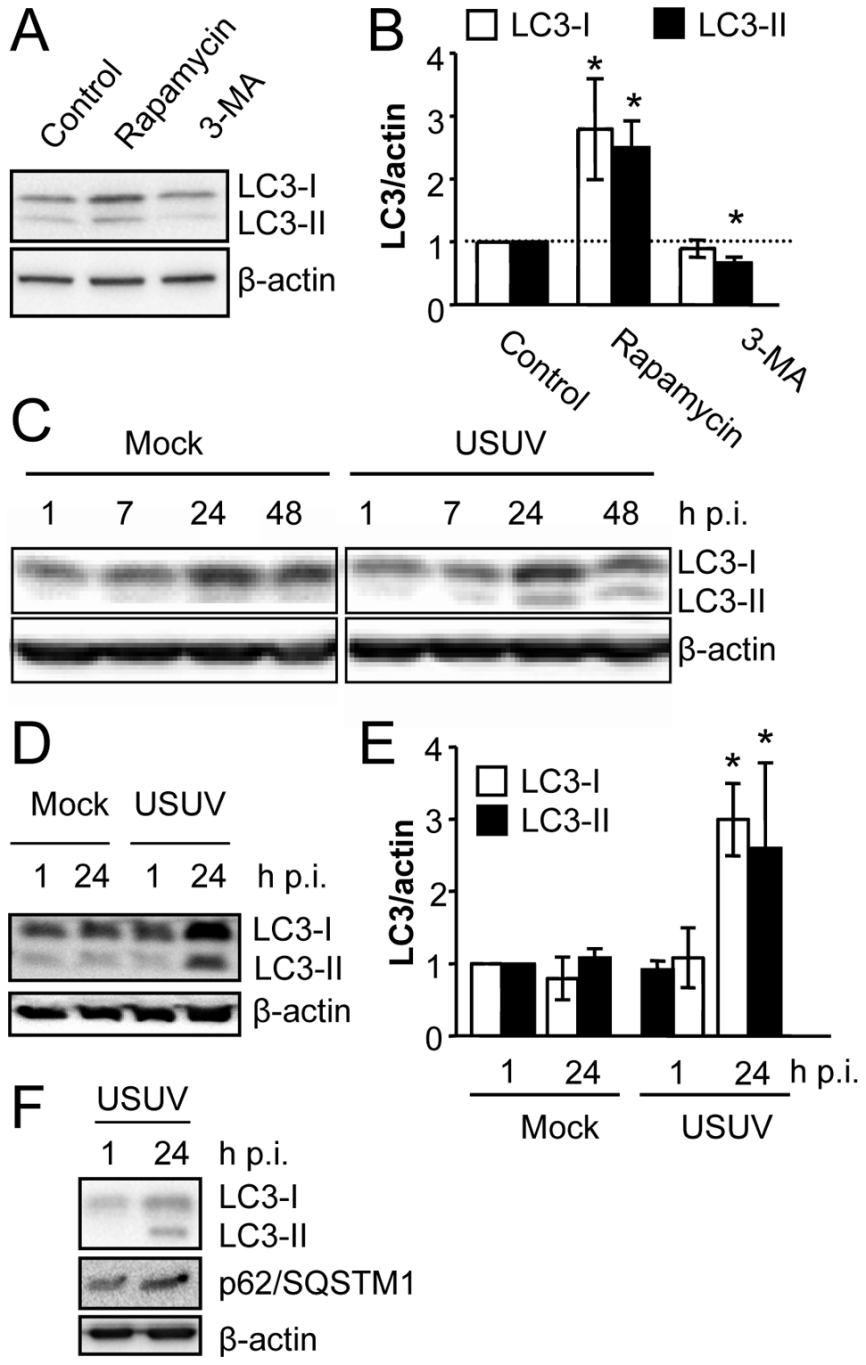
Analysis of LC3 modification following infection by USUV. (A) Changes in LC3 levels induced by pharmacological modulation of autophagy. Vero cells were treated with either 3-MA or rapamycin for 24 h. Cells were lysed and LC3 was detected by western blotting using specific antibodies. Membrane was reincubated with an anti-β-actin antibody as a control for protein loading. (B) Quantification of LC3 species (LC3-I and LC3-II) by densitometry of western blots performed as in (A). Data were normalized across the experiments relative to control cells. (C) Cells infected or not with USUV SAAR 1776 (MOI of 0.5 PFU/cell) were lysed at different times p.i. and subjected to western blot analysis using an antibody against LC3 to detect non-lipidated LC3 (LC3-I) and LC3 conjugated to phosphatidylethanolamine (LC3-II). (D) Similar analysis to that performed in (C) was carried using a PVDF membrane instead of the nitrocellulose membrane used in (C). (E) Quantification of LC3 species (LC3-I and LC3-II) by densitometry of western blots of cells infected in (D). Data were normalized across the experiments relative to mock-infected cells. (F) Analysis of p62/SQSTM1 levels on cells infected with USUV SAAR 1776. The levels of p62/SQSTM1 were determined by western blot on Vero cells infected as in (B). LC3 and β-actin are also shown. Statistically significant differences are denoted by one asterisk (*) for P<0.05.

Following induction of autophagy, microtubule-associated protein 1 light chain 3 (LC3), a mammalian homolog of yeast Atg8 (autophagy-related protein 8) is conjugated to phosphatidylethanolamine and targeted to autophagic membranes labelling autophagic vacuoles [34], [40]. This prompted us to analyze LC3 modification following infection with USUV. As controls, Vero cells treated in parallel with the inhibitor of autophagy 3-methyladenine (3-MA) or with the inductor of autophagy rapamycin [40] were analyzed by western blot (Fig. 2A). A significant increase in LC3-II was noticed on cells treated with rapamycin, whereas a significant decrease of LC3-II was observed in cells treated with 3-MA (Fig. 2A). Quantification of the LC3-II/actin ratio confirmed these observations (Fig. 2B). Interestingly, a significant increase in LC3-I was also noticed in cells treated with rapamycin, suggesting that rapamycin could not only promote LC3-I/II turnover whereas it could also result in accumulation of both LC3 species under our experimental conditions. As these results confirmed the reliability of western blot assays to detect alterations of the autophagic flux, the modification of LC3 was analyzed on cells infected with USUV (Fig. 2C). An increase in the amount of LC3-II was observed following infection with USUV when compared to mock-infected cells. This finding is compatible with alterations of the autophagic pathway in cells infected with USUV. Since PVDF membranes can result more sensitive to detect LC3-II than those of nitrocellulose [41], we performed similar analyses using these membranes. An increase in both LC3-I and LC3-II along time was observed in samples infected with USUV (Fig. 2D), which was confirmed by densitometry of protein bands (Fig. 2E), as commented above for cells treated with rapamycin. The accumulation of total LC3 (LC3-I and LC3-II) suggests that the formation of autophagosomes could be upregulated in USUV infected cells. Indeed, accumulation of total LC3 has been documented during DENV-induced autophagy [24]. As the increase in LC3-II was not accompanied by a decrease in LC3-I, this could indicate that the autophagic flux, and hence LC3-I/II turnover, is not upregulated in cells infected with USUV, or that expression of LC3 is stimulated by USUV infection. In fact, transcription of LC3 is increased in certain systems upon induction of autophagy [41]. To analyze if USUV infection altered the normal autophagic flux that involves degradation or turnover of autophagosomal proteins, the levels of other autophagy-related protein, p62/SQSTM1, were analyzed in USUV infected cells. The degradation of p62/SQSTM1, a polyubiquitin-binding protein that interacts with LC3 [35], has been described following upregulation of the autophagic flux under certain conditions [40]. However, no change of p62/SQSTM1 levels was found in cells infected with USUV (Fig. 2F), These results indicate that Vero cells infected with USUV presented an accumulation of both LC3-II and LC3-I, a finding that could suggest an accumulation of autophagosomes, apparently not associated to alteration of p62/SQSTM1 level.

### Infection with USUV increases autophagosome formation

**Figure 3:**
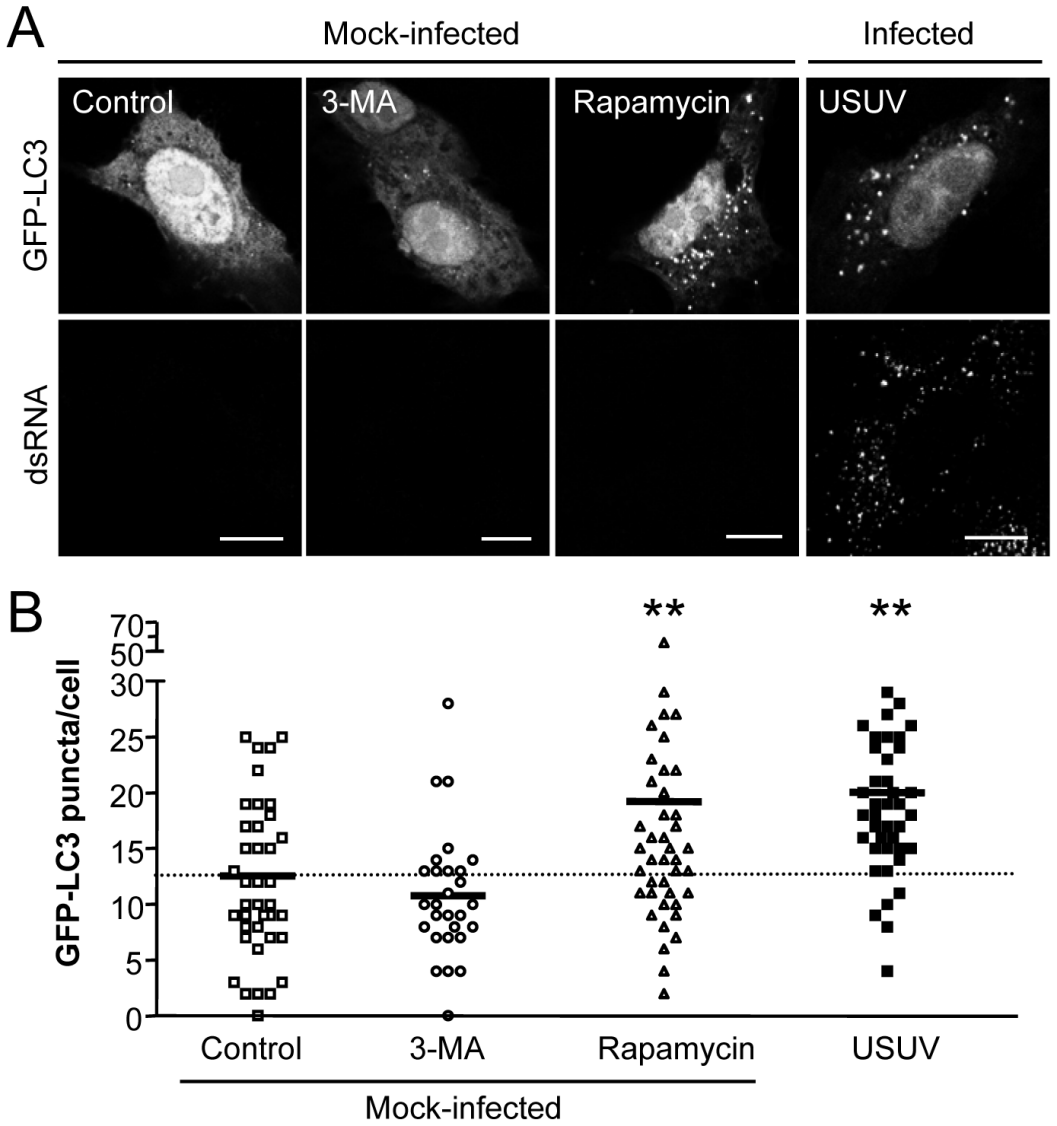
Induction of LC3 aggregation on cells infected with USUV. (A) Visualization of autophagosome formation by LC3 aggregation in cells infected with USUV. Vero cells were transfected with a plasmid encoding GFP-LC3 and 24 h post-transfection were infected with USUV SAAR 1776 (MOI of 5 PFU/cell), or treated with 3-MA or rapamycin as controls. Cells were fixed and processed for immunofluorescence using a monoclonal antibody against dsRNA and secondary antibodies AF-594 labelled 24 h p.i. Scale bars: 10 µm. (B) Quantification of the number of LC3 aggregates per cell. The number of fluorescent aggregates on the cytoplasm of cells treated as in (A) was determined. Each point in the graph represents a different cell. Solid lines represent the mean number of GFP puncta per condition. Dashed line indicates the mean number of GFP puncta aggregates found in control cells. Statistically significant differences between each condition and control cells are denoted by two asterisks (**) for P<0.005.

As commented above, following induction of autophagy the lipidated form of LC3 is targeted to autophagic membranes labelling autophagic vacuoles [34], [40]. This feature can be monitored by fluorescence microscopy as an increase in LC3 puncta in the cell cytoplasm [34], [40]. Vero cells were transfected with a plasmid encoding GFP-LC3 for 24 h, and then infected with USUV (Fig. 3A). Cells were fixed at 24 h p.i. and samples were processed for immunofluorescence using an antibody specific for double-stranded RNA (dsRNA) -a well characterized marker of the flavivirus replication complex [32], [37], [38]- to verify that transfected cells were infected. As expected, no positive signal corresponding dsRNA was observed on mock-infected cells, whereas fluorescent spots could be detected in the cytoplasm of USUV-infected cells (Fig. 3A). As controls, transfected cells were also treated in parallel with 3-MA or with rapamycin. Cells treated with rapamycin displayed a spotted cytoplasm compatible with the formation of LC3 aggregates as a result of the upregulation of autophagy, whereas cells treated with 3-MA were apparently undistinguishable from control cells (Fig. 3A). The cytoplasmic aggregates displayed by cells infected with USUV suggest an upregulation of the autophagic pathway during USUV-infection. When the number of puncta corresponding to GFP-LC3 per cell was determined by fluorescence microscopy [40] (Fig. 3B), it was found that cells treated with 3-MA displayed a tendency to a reduction on the number of LC3 aggregates, although not statistically significant, whereas a statistically significant increase was observed in rapamycin treated cells, thus validating the reliability of this method to detect induction of autophagy. A statistically significant increase in the mean number LC3 aggregates in cells infected with USUV was also noticed. The extent of this increase was similar to that found for cells treated with rapamycin. Therefore, this analysis confirmed the accumulation of autophagosomes in cells infected with USUV.

### Autophagosomes are not the place for USUV replication

**Figure 4:**
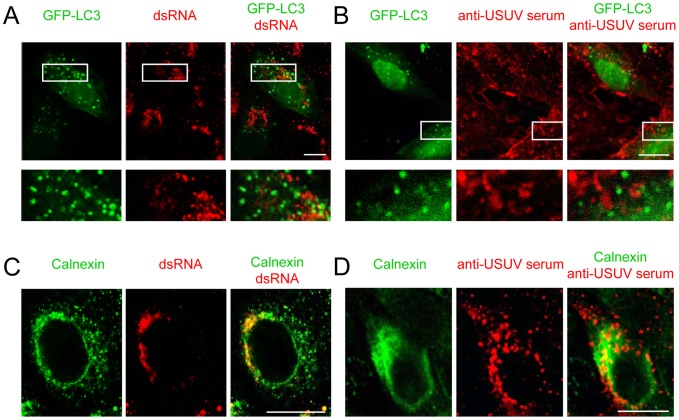
Replication of USUV does not take place on autophagosomes. (A) Representative confocal image of Vero cells transfected with GFP-LC3 plasmid and infected with USUV SAAR 1776 (MOI of 5 PFU/cell) 24 h post-transfection. Cells were fixed and processed for immunofluorescence 24 h post-infection using a monoclonal antibody against dsRNA and secondary antibodies AF-594 labelled. GFP-LC3 is shown green and dsRNA in red. (B) Cells transfected and infected as in (A) were stained with a polyclonal serum from a mouse experimentally infected with USUV to detect viral proteins. GFP-LC3 is shown in green and USUV proteins in red. (C) Cells infected as in (A) were immunostained using rabbit antibody against calnexin and a monoclonal antibody against dsRNA. Calnexin is shown in green and dsRNA in red. (D) Cells infected as in (A) were immunostained using a rabbit antibody against calnexin and the polyclonal mouse serum against USUV. Calnexin is shown in green and USUV proteins in red. Scale bars: 10 µm.

It has been proposed that replication of several members of the *Flaviviridae* family may be based on autophagosome membranes labelled with LC3 [28], [42], although other studies do not support this view [21], [43]. Detailed observation of USUV-infected cells revealed that GFP-LC3 puncta did not colocalize with dsRNA positive spots that labelled USUV RNA replication sites (Fig. 4A), thus indicating that although USUV infection increased the formation of autophagosomes the viral replication did not take place directly on autophagic membranes. The localization of viral proteins in infected cells was analyzed using a specific polyclonal serum raised against USUV (Fig. S1). As commented for dsRNA, USUV proteins did not colocalize with GFP-LC3 aggregates (Fig. 4B), pointing that viral proteins were not associated with autophagic membranes. In contrast to this, dsRNA colocalized with calnexin (Fig. 4C), a maker from the endoplasmic reticulum, a finding consistent with previous observations pointing that replication of USUV is based on membrane structures derived from the endoplasmic reticulum [32]. The viral proteins stained with the polyclonal serum also partially colocalized with calreticulin (Fig. 4D), thus confirming the interaction of the endoplasmic reticulum and viral components during USUV infection.

### Acidification status of LC3 aggregates induced by USUV

**Figure 5:**
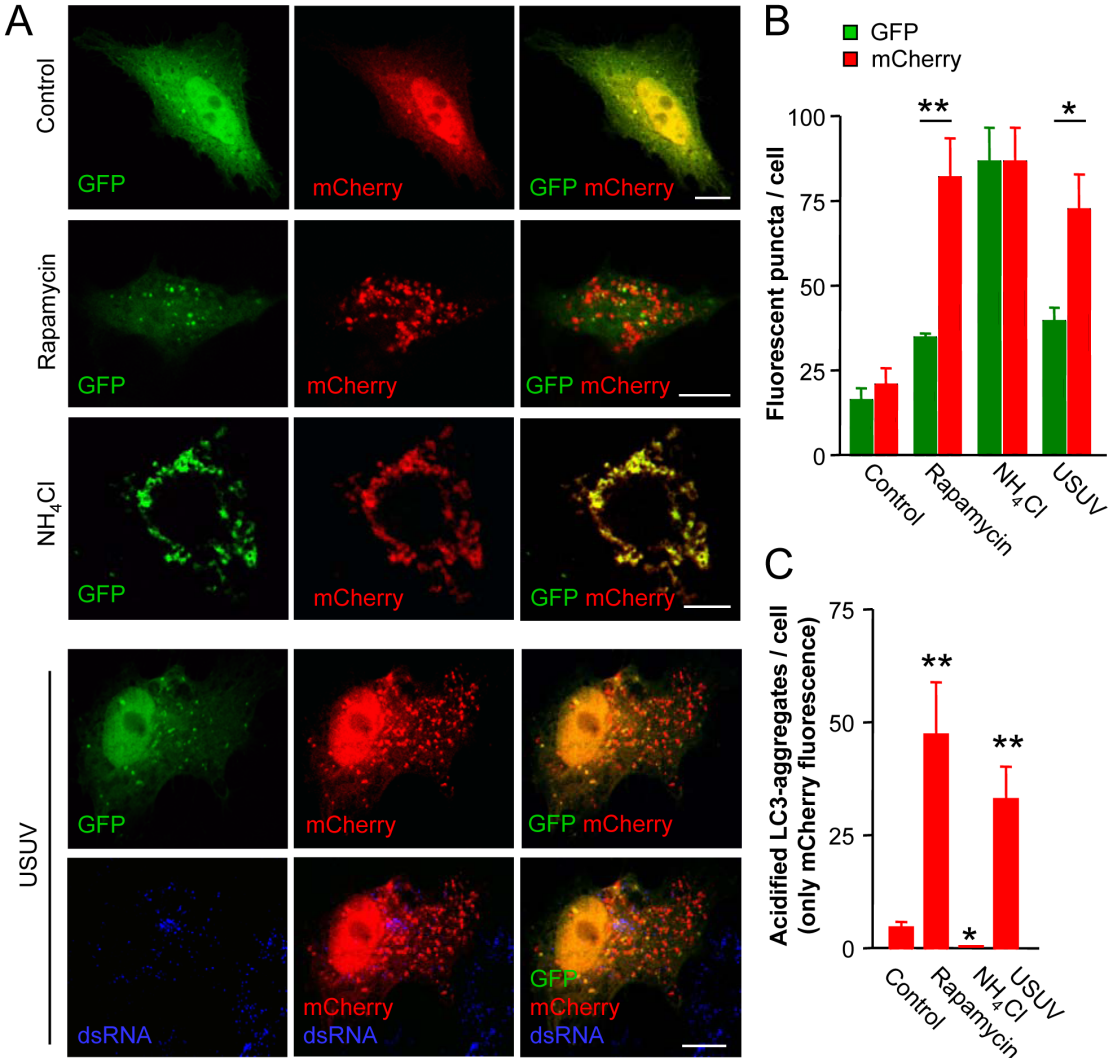
Accumulation of acidified autophagosomal structures in USUV-infected cells. (A) Vero cells were transfected with mCherry-GFP-LC3 plasmid and treated with rapamycin, NH_4_Cl or infected with USUV SAAR 1776 (MOI of 5 PFU/cell) 24 h post-transfection. Cells were fixed and processed for immunofluorescence (24 h p.i. or after treatment with rapamycin or NH_4_Cl) using monoclonal antibody to detect dsRNA and appropriated Alexa Fuor-647 secondary antibodies. Colocalization between GFP and mCherry denotes autophagosomal structures that have not already fused with a lysosomal compartment (phagophores or autophagosomes) whereas mCherry signal without GFP signal denotes autophagosomal acidified organelle that have fused with lysosomes (amphisomes or autolysosomes). GFP, in green; mCherry, in red; dsRNA, in blue. Scale bars: 10 µm. (B) Quantification of the number of fluorescent puncta exhibiting green (GFP) or red (mCherry) fluorescence in cells transfected with mCherry-GFP-LC3 plasmid and treated or infected as in (A). Statistically significant differences between the number of red and green puncta displayed by the cells are denoted by one asterisk (*) for P<0.05 or two asterisks (**) for P<0.005. (C) Quantification of the number of fluorescent puncta displaying only mCherry fluorescence (acidified autophagosomal structures) of cells transfected with mCherry-GFP-LC3 plasmid and treated or infected as in (A). Statistically significant differences between each condition and control cells are denoted by one asterisk (*) for P<0.05 or two asterisks (**) for P<0.005.

Since GFP is acid-labile, it makes difficult to detect autophagosomal structures by fluorescence microscopy using GFP-LC3 once they have fused with endosomes or lysosomes [35]. To analyze if USUV-infected cells were enriched only on autophagosomal compartments that had not already fused with acidic compartments we used a tandem mCherry-GFP-tagged LC3 expression vector [35]. The mechanism of action of this construction is based on that GFP signal is reduced in an acidic environment, whereas mCherry is more stable [35], [40], [41]. In this way, colocalization of GFP and mCherry indicates a cellular compartment that has not fused with an acidic compartment (phagophore or autophagosome) whereas mCherry signal without GFP corresponds to an autophagosomal compartment that has fused with an endosome or lysosome (amphisome or autolysosome) [40]. To verify that this construction worked properly under the experimental settings, cells were transfected with mCherry-GFP-tagged LC3 plasmid and then treated with rapamycin (to promote autophagosome maturation) or with NH_4_Cl (to block the normal autophagic flux) (Fig. 5A) [41], [44]. When compared to control cells, an increase in the number of GFP puncta following rapamycin treatment was observed, however the increase in mCherry puncta was more marked (Fig. 5B), indicating that rapamycin promoted maturation of autophagosomal structures towards acidic organelles in which GFP fluorescence was lost although they retained mCherry fluorescence. In contrast, the impairment of organelle acidification and the blockage of the normal autophagic flux exerted by NH_4_Cl induced an accumulation of LC3 puncta positive for both GFP and mCherry (Fig. 5A and B) that indicated a reduction in the number of acidified autophagic structures (Fig. 5C). Cells infected with USUV displayed an accumulation of mCherry puncta that did not colocalize with GFP puncta (Fig. 5A, B and C). This indicates that at this time postinfection there is an accumulation of acidified autophagosomal structures in USUV-infected cells that correspond to amphisomes or autolysosomes. In addition to this, no colocalization between dsRNA and mCherry was found in these experiments indicating that neither autophagosomes nor autolysosomes are the places for replication of USUV (Fig. 5A). This is again consistent with the notion that replication of USUV is associated to the endoplasmic reticulum and not to structures of an autophagic origin.

### Activation of the unfolded protein response following infection with USUV

**Figure 6:**
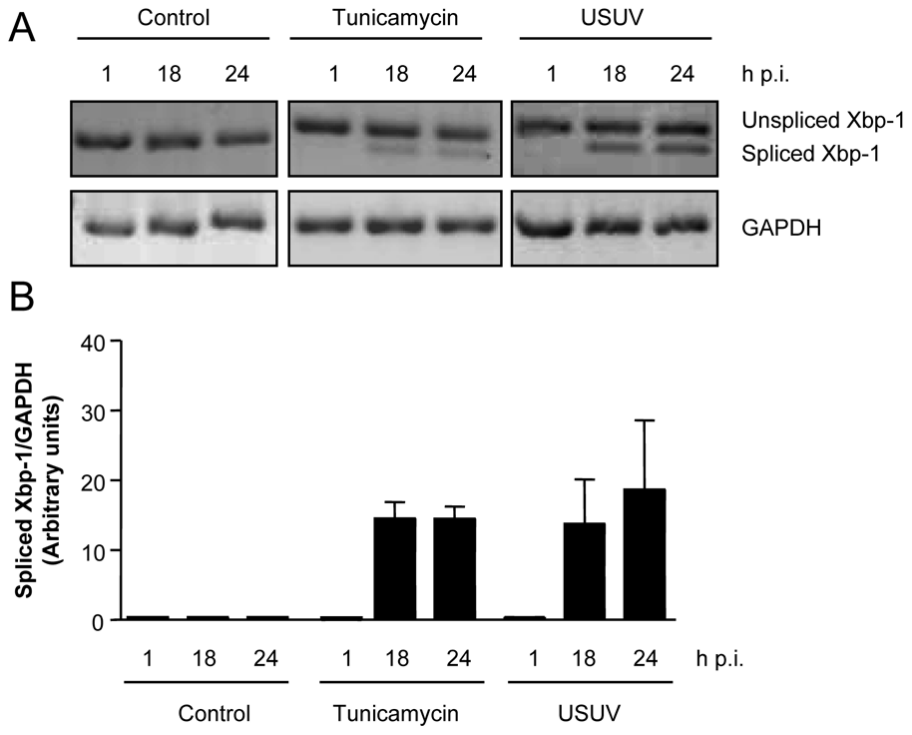
USUV-infection activates the unfolded protein response. (A) RNA was extracted from Vero cells infected with USUV SAAR 1776 (MOI of 0.5 PFU/cell) at different time p.i. and the presence of unspliced or spliced Xbp-1 mRNA was determined by RT-PCR. Cells treated with tunicamycin are included as a positive control of the activation of unfolded protein response. GAPDH mRNA was also amplified by RT-PCR as a control. (B) Quantification of the intensity of the band corresponding to amplified spliced Xbp-1 in cells treated as in (A) normalized by the intensity of amplified GAPDH.

The interaction of flavivirus with endoplasmic reticulum during viral replication can result in induction of endoplasmic reticulum stress [45], [46], [47]. To cope with this problem, flavivirus-infected cells can undergo a coordinated change in gene expression collectively known as unfolded protein response [45], [46], [47]. Since induction of the unfolded protein response can trigger an autophagic response [48], we analyzed if infection by USUV also activated the unfolded protein response. To this end, we monitored the splicing of Xbp-1 (X box binding protein 1) mRNA (Fig. 6A), which allows expression of the full length transcription factor Xbp-1 that upregulates transcription of multiple genes aimed to cope with endoplasmic reticulum stress and that has been detected as a common feature of unfolded protein response in flavivirus-infected cells [45]. Cells treated with tunicamycin, to pharmacologically induce the unfolded protein response, displayed an increase in the amount of spliced Xbp-1 not observed in control cells. Cells infected with USUV also displayed an increase in the amount of spliced Xbp-1 that was observed between 18 and 24 h p.i. The amount of spliced Xbp-1 detected in USUV-infected cells was comparable to that observed in tunicamycin treated cells (Fig. 6B). These results evidenced that infection with USUV shared in common with other flaviviruses the activation of the unfolded protein response.

### Effects of pharmacological modulation of the autophagic pathway on USUV infection

**Figure 7:**
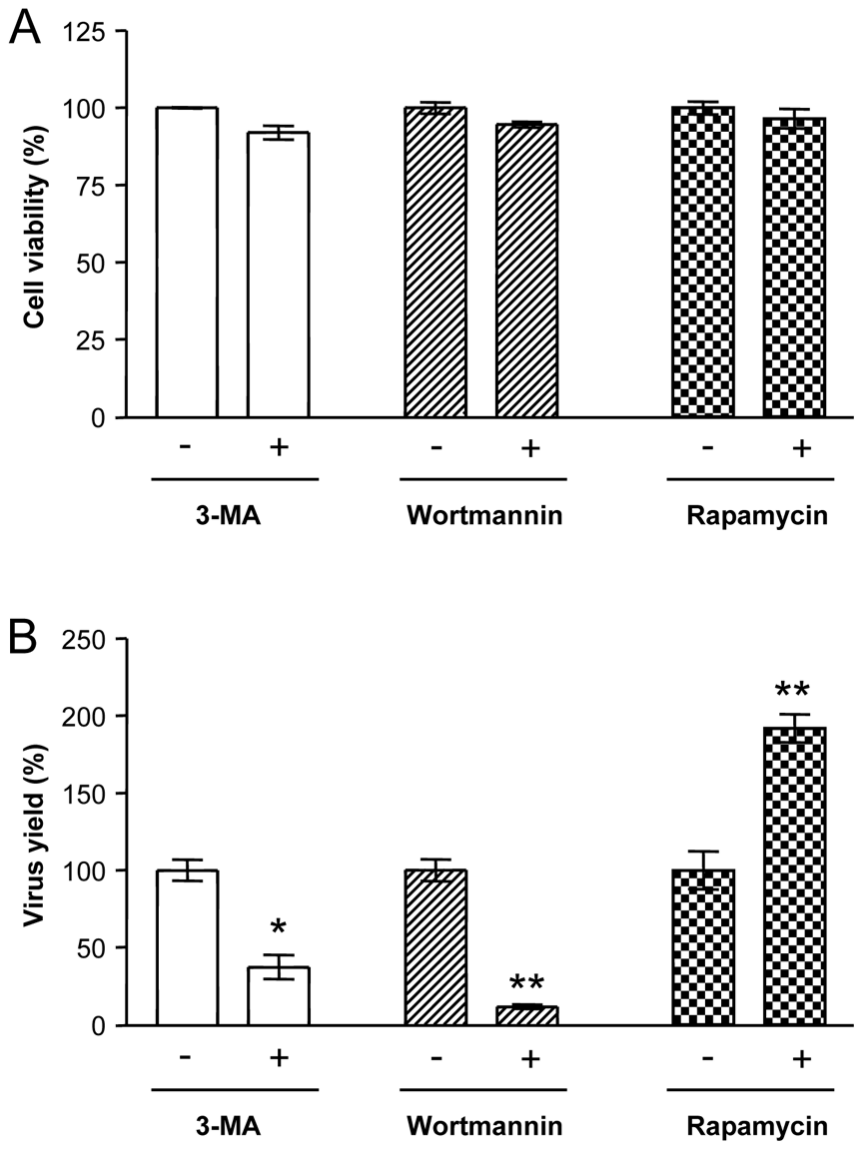
Effects of pharmacological modulation of autophagy on USUV infection. (A) Analysis of cellular viability upon treatment with 3-MA, wortmannin or rapamycin. Vero cells were treated with the drugs for 24 h and the viability was estimated by determination of cellular ATP levels by a luminescence assay. (B) Effect of pharmacological modulation of autophagy on USUV production. Cells infected with USUV SAAR 1776 (MOI of 0.5 PFU/cell) were treated with 3-MA, wortmannin or rapamycin and the virus yield were determined by plaque assay at 24 h p.i. Statistically significant differences between each condition and control cells are denoted by one asterisk (*) for P<0.05 or two asterisks (**) for P<0.005.

Overall, our results pointed to an upregulation of the autophagic pathway in USUV-infected cells and, thus, the possibility of manipulating the autophagic pathway to modulate infection with USUV was addressed. Hence, to evaluate the potential of pharmacological modulation of autophagy as a candidate for antiviral approach design against USUV, the effect of two inhibitors of autophagy, 3-MA and wortmannin [49], and that of the inductor of autophagy rapamycin were analyzed [40]. First of all, the cellular viability under drug-treatments was determined (Fig. 7A). After 24 h of treatment with 3-MA, wortmannin, or rapamycin no major toxic effects on Vero cells were noticed, confirming the adequacy of these conditions for subsequent analyses. Treatment with either 3-MA or wortmannin resulted in a significant reduction of the virus yield of USUV virus (Fig. 7B). On the other hand, rapamycin induced a significant increase in the viral production of USUV. Taken together, these observations support an implication of the autophagic machinery on the replication of USUV and confirm that pharmacological intervention on the autophagic pathway modulates USUV infection.

### Autophagic response in cells infected with a recent European USUV isolate

**Figure 8:**
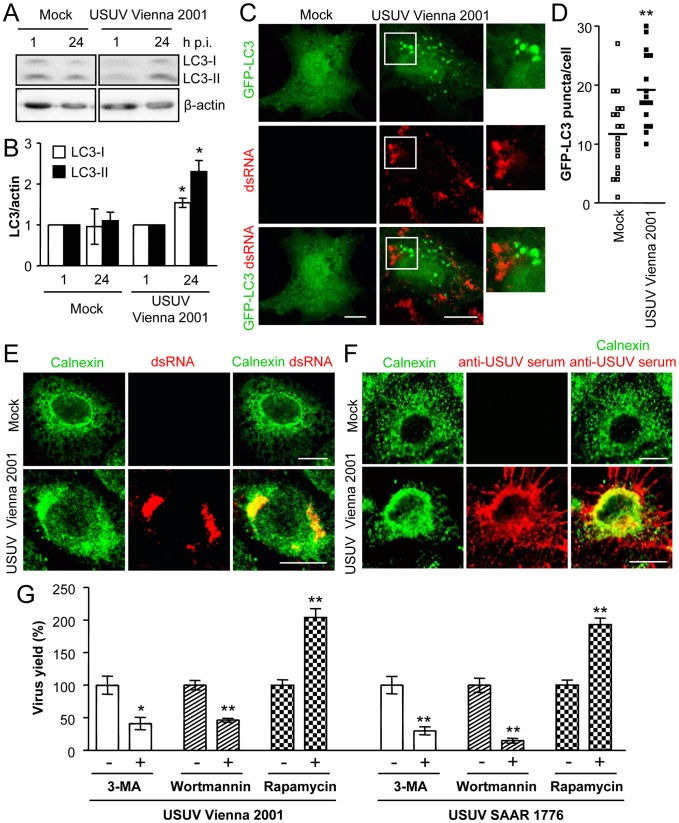
Autophagic features induced by the infection of a recent European isolate of USUV. (A) Analysis of LC3 modification following infection with USUV Vienna 2001. Vero cells infected or not with USUV Vienna 2001 (MOI of 0.5 PFU/cell) were lysed at different times p.i. and subjected to western blot analysis using a PVDF membrane and an antibody against LC3 to detect non-lipidated LC3 (LC3-I) and LC3 conjugated to phosphatidylethanolamine (LC3-II). Membrane was reincubated with an anti-β-actin antibody as a control for protein loading. (B) Quantification of LC3 species by densitometry of western blots performed as in (A) normalized across the experiments. (C) Visualization of autophagosome formation by LC3 aggregation in cells infected with USUV Vienna 2001. Vero cells were transfected with a plasmid encoding GFP-LC3 and 24 h post-transfection were infected with USUV Vienna 2001 (MOI of 5 PFU/cell). Cells were fixed and processed for immunofluorescence using a monoclonal antibody against dsRNA and secondary antibody AF-594 labelled 24 h p.i. (D) Quantification of the number of LC3 aggregates per cell. The number of fluorescent aggregates on the cytoplasm of cells infected as in (C) was determined. Each point in the graph represents a different cell. Solid lines represent the mean number of GFP puncta per condition. (E) Cells infected as in (B) were immunostained using rabbit antibody against calnexin and a monoclonal antibody against dsRNA. Calnexin is shown in green and dsRNA in red. (F) Cells infected as in (A) were immunostained using rabbit antibody against calnexin and the polyclonal mouse serum against USUV. Calnexin is shown in green and USUV proteins in red. (G) Effect of pharmacological modulation of autophagy on USUV Vienna 2001 production. Cells infected with USUV Vienna 2001 or SAAR 1776 (MOI of 0.5 PFU/cell) were treated with 3-MA, wortmannin or rapamycin and the virus yield were determined by plaque assay at 24 h p.i. Statistically significant differences between each condition and control cells are denoted by one asterisk (*) for P<0.05 or two asterisks (**) for P<0.005. Scale bars: 10 µm.

The findings reported above were obtained using the prototypic USUV strain SAAR 1776 whose pathogenic capability has not been conclusively proven even in the birds. To analyze if the USUV circulating in Europe displayed similar interactions with the autophagic pathway, the USUV strain Vienna 2001 (a recent isolate of USUV which pathogenicity has been extensively proven at least in birds) was included in the study [6], [50]. Infection with USUV Vienna 2001 induced an increase in the levels of LC3-II and also LC3-I, as described for USUV SAAR 1776 (Fig. 8A and B). In addition to this, cells transfected with the plasmid encoding GFP-LC3 and infected with USUV Vienna 2001 also displayed a significant accumulation of GFP-LC3 aggregates throughout the cytoplasm compared to mock-infected cells (Fig. 8C and D). These fluorescent GFP-LC3 aggregates did not colocalize with dsRNA (Fig. 8C) as commented for cells infected with USUV SAAR 1776 (Fig. 4A). However, dsRNA colocalized with the endoplasmic reticulum marker calnexin (Fig. 8E) as described for USUV SAAR 1776 (Fig. 4C). Even more, USUV proteins detected using a specific mouse serum colocalized with calnexin (Fig. 8F) confirming the association of viral antigens of USUV Vienna 2001 with endoplasmic reticulum, as described for USUV SAAR 1776 (Fig. 4D). The effect of the inhibitors of autophagy 3-MA and wortmannin, and that of the inductor of autophagy rapamycin was also analyzed in parallel for USUV Vienna 2001 and SAAR 1776 (Fig. 8G). Treatment with 3-MA or wortmannin resulted in a significant reduction of the virus yield of USUV Vienna 2001 as well as USUV SAAR 1776. The extent of inhibition exerted by 3-MA was similar for both viral strains, whereas USUV Vienna 2001 was slightly less inhibited by wortmannin than USUV SAAR 1776. In contrast, rapamycin induced a significant increase in the viral production of both USUV strains. Taken together, these results indicate that the findings observed for USUV SAAR 1776 were shared by USUV Vienna 2001, a strain of USUV that is currently circulating in Europe with documented pathogenecity in birds.

## Discussion

The *Flavivirus* genus comprises more than 50 viral species that include well long known arthropod-borne pathogens as DENV, WNV, JEV, St. Louis encephalitis virus, Murray Valley encephalitis virus, Yellow fever virus or tick-borne encephalitis virus (http://www.ictvonline.org/virusTaxonomy.asp?version=2012). But this viral genus also contains other neglected viral pathogens of currently increasing interest. Among these recently considered potential threats is USUV, a flavivirus endemic from Africa that emerged in Europe during the last decade (see Introduction).

In addition to basic knowledge, characterization of cellular pathways involved in virus replication could help to identify novel therapeutic targets. In this regard, the interaction of USUV with the host cell almost remains as an unexplored field, so at this point the identification of cellular pathways that regulate USUV infection is desirable. In this study we have explored the possible interaction of USUV with the autophagic pathway during infection. Due to the availability of more suitable reagents to analyze the autophagic pathway in mammalian cells, we selected Vero cells for the analysis. In fact, Vero cells constitute a cell line widely used for the cultivation and titration of USUV. In this way, the interaction of USUV with the autophagic pathway in cells derived from bird or mosquito, the main natural hosts for USUV, remains to be further evaluated. Our results showed that infection in mammalian cells by either the reference South African strain of USUV (SAAR 1776) or a recent European strain (Vienna 2001) triggered an autophagic response in the host cell. This is consistent with findings obtained for other flaviviruses [21], [23], [24], [25], [26]. The autophagic response was characterized by an increase in the levels of both LC3-II and LC3-I, which correlated with the accumulation of autophagic structures in the cytoplasm of infected cells. Our results also showed an induction of Xbp-1 mRNA splicing following USUV infection. Xbp-1 mRNA splicing constitutes a marker of the induction of the unfolded protein response, a finding that has been related to autophagic process during the viral infection [51]. Regarding the characteristics of the autophagic response induced by USUV, no p62/SQSTM1 degradation was found in infected cells, a feature that has been also described for other flaviviruses [26], [29]. This finding together with the accumulation of both LC3-I and LC3-II could suggest an incomplete autophagic response that takes place without autophagosome maturation. However, the experiments performed with mCherry-GFP-LC3 plasmid revealed that USUV-infected cells were enriched in acidified autophagosomal structures, suggesting that at least a significant proportion of autophagic structures can maturate and fuse with acidified organelles in USUV-infected cells. These structures could include amphisomes or autolysosomes, whose morphology is compatible with those of multi-lamellar organelles observed by transmission electron microscopy [39]. Regarding the accumulation of both LC3-I and LC3-II, this has been described as a feature of DENV-induced autophagy [24], and was also observed for cells treated with rapamycin. Although this could result of reduced autophagosomal degradation, the detection of acidified autophagosomal structures in USUV-infected suggests that autophagosomes can maturate in USUV-infected cells. However, another non-excluding possibility is an increase of expression of LC3 following infection of USUV, since such increase by a mechanism involving the unfolded protein response has been documented [41], and this response is also activated following USUV infection. According with this possibility, the increase in other cellular proteins involved in autophagy, as p62/SQSTM1, during infection with the related flavivirus WNV has also been proposed [29].

There is a controversy related to the autophagic origin or not of the structures that provide the platform for replication of distinct members of the *Flaviviridae* family as DENV or HCV. While several studies have been pointed that viral replication may be based on membranes of autophagosomal origin that contain LC3 [28], [42] other studies clearly contradict these results [21], [43]. In USUV-infected cells, no major colocalization between LC3 containing structures and dsRNA (a well characterized marker of the flavivirus replication complex) was found. In addition to this, no colocalization was found between USUV proteins and LC3, indicating that viral proteins were not associated with autophagic structures, and suggesting that these structures did not provide the main platform for viral replication. In fact, these results agree with data pointing that USUV replication, as well as those of WNV or DENV [37], [38], is mainly based on modified endoplasmic reticulum structures [37], [38]. Even more, dsRNA in USUV infected cells colocalized with calnexin, a marker of the endoplasmic reticulum. In this way, USUV replication most probably would take place associated to the vesicle packets (VPs), which in other viral models have been shown to contain the dsRNA intermediates and have been probed to be constituted by invaginations of endoplasmic reticulum-derived membranes [37], [38]. Even more, the observed colocalization between calnexin and USUV proteins confirms the interaction of viral components with the endoplasmic reticulum.

Autophagy constitutes a major metabolic pathway that is currently being explored for treatment of multiple human disorders that include certain types of cancer and metabolic diseases, neurological disorders or viral infections [14], [16], [22]. In this regard, drugs that interfere with the autophagic pathway were assayed. The inductor of autophagy rapamycin increased virus yield of both USUV strains analyzed. In contrast to this, two structurally unrelated inhibitors of phosphatidylinositol 3-kinases (PI3Ks) involved in the induction of autophagy (3-MA and wortmannin) decreased virus yield of both USUV strains here analyzed, including the European USUV isolate Vienna 2001, representative of the USUV that is currently circulating in Europe. Interestingly, whereas 3-MA inhibited both viral strains in a similar manner, infection by USUV Vienna 2001 was less inhibited by wortmannin than that of USUV SAAR 1776. These observations point to the autophagic pathway as a novel partner of USUV infection and specifically point to PI3Ks as valid antiviral targets. Having in mind that different PI3Ks are under strict consideration as cellular targets for treatment of human disorders [52], the results here presented set a starting point for antiviral development to combat USUV based on inhibition of these cellular enzymes. Other previously identified cellular pathways as regulators of USUV infection have been the synthesis of fatty acids [32], the innate immune response induced in infected cells [53], and preliminary data also point to the induction of apoptosis in infected cells [32]. In fact, connections between autophagy and these other metabolic pathways involved on USUV infection have been also documented for other members of the *Flaviviridae* family [21], [24], [54]. In this way, the identification of the involvement of autophagy during USUV infection will help to decipher the puzzle of the interaction of USUV with host cells.

Overall this study provides the first evidence for a role of autophagy during the infection of the mosquito-borne USUV. Our results indicate that pharmacological inhibition of the autophagic pathway can reduce infection by this virus in cultured cells. These observations identify autophagy as a metabolic pathway involved on USUV-infection, thus opening a potential new research line for the design of antiviral therapies against this pathogen.

## Supporting Information

Figure S1 **Specific staining of USUV-infected cells with a polyclonal serum.** Vero cells were infected or not (mock) with USUV SAAR 1776 (MOI of 5 PFU/cell) and fixed and processed for immunofluorescence (24 h p.i.) using the serum obtained from a mouse experimentally infected with USUV. AF-488 anti-mouse IgG was used as secondary antibody. Both images were acquired using the same microscope settings. Scale bars: 10 µm.
(TIF)

## References

[1] Kilpatrick AM (2011) Globalization, land use, and the invasion of West Nile virus. Science 334: 323–327. 22021850 10.1126/science.1201010 PMC3346291

[2] Weaver SC, Reisen WK (2010) Present and future arboviral threats. Antiviral Res 85: 328–345. 19857523 10.1016/j.antiviral.2009.10.008 PMC2815176

[3] Brault AC (2009) Changing patterns of West Nile virus transmission: altered vector competence and host susceptibility. Vet Res 40: 43. 19406093 10.1051/vetres/2009026 PMC2695027

[4] Vazquez A, Jimenez-Clavero M, Franco L, Donoso-Mantke O, Sambri V, et al (2011) Usutu virus: potential risk of human disease in Europe. Euro Surveill 16: pii = 19935. 21871214

[5] Nikolay B, Diallo M, Boye CS, Sall AA (2011) Usutu virus in Africa. Vector Borne Zoonotic Dis 11: 1417–1423. 21767160 10.1089/vbz.2011.0631

[6] Weissenbock H, Kolodziejek J, Url A, Lussy H, Rebel-Bauder B, et al (2002) Emergence of Usutu virus, an African mosquito-borne flavivirus of the Japanese encephalitis virus group, central Europe. Emerg Infect Dis 8: 652–656. 12095429 10.3201/eid0807.020094 PMC2730324

[7] Gaibani P, Pierro A, Alicino R, Rossini G, Cavrini F, et al (2012) Detection of Usutu-virus-specific IgG in blood donors from northern Italy. Vector Borne Zoonotic Dis 12: 431–433. 22217176 10.1089/vbz.2011.0813

[8] Allering L, Jost H, Emmerich P, Gunther S, Lattwein E, et al (2012) Detection of Usutu virus infection in a healthy blood donor from south-west Germany, 2012. Euro Surveill 17: 20341. 23241231

[9] Savini G, Monaco F, Terregino C, Di Gennaro A, Bano L, et al (2011) Usutu virus in Italy: an emergence or a silent infection? Vet Microbiol 151: 264–274. 21550731 10.1016/j.vetmic.2011.03.036

[10] Lupulovic D, Martin-Acebes MA, Lazic S, Alonso-Padilla J, Blazquez AB, et al (2011) First serological evidence of West Nile virus activity in horses in Serbia. Vector Borne Zoonotic Dis 11: 1303–1305. 21438694 10.1089/vbz.2010.0249

[11] Pecorari M, Longo G, Gennari W, Grottola A, Sabbatini A, et al (2009) First human case of Usutu virus neuroinvasive infection, Italy, August–September 2009. Euro Surveill 14: pii = 19446. 20070936

[12] Cavrini F, Gaibani P, Longo G, Pierro AM, Rossini G, et al (2009) Usutu virus infection in a patient who underwent orthotropic liver transplantation, Italy, August–September 2009. Euro Surveill 14: pii: 19448. 20070935

[13] Orvedahl A, Levine B (2008) Viral evasion of autophagy. Autophagy 4: 280–285. 18059171 10.4161/auto.5289 PMC3508671

[14] Mizushima N, Levine B, Cuervo AM, Klionsky DJ (2008) Autophagy fights disease through cellular self-digestion. Nature 451: 1069–1075. 18305538 10.1038/nature06639 PMC2670399

[15] Yordy B, Tal MC, Hayashi K, Arojo O, Iwasaki A (2012) Autophagy and selective deployment of Atg proteins in antiviral defense. Int Immunol 25: 1–10. 23042773 10.1093/intimm/dxs101 PMC3534236

[16] Levine B, Kroemer G (2008) Autophagy in the pathogenesis of disease. Cell 132: 27–42. 18191218 10.1016/j.cell.2007.12.018 PMC2696814

[17] Joubert PE, Werneke SW, de la Calle C, Guivel-Benhassine F, Giodini A, et al (2012) Chikungunya virus-induced autophagy delays caspase-dependent cell death. J Exp Med 209: 1029–1047. 22508836 10.1084/jem.20110996 PMC3348111

[18] Jackson WT, Giddings TH Jr, Taylor MP, Mulinyawe S, Rabinovitch M, et al (2005) Subversion of cellular autophagosomal machinery by RNA viruses. PLoS Biol 3: e156. 15884975 10.1371/journal.pbio.0030156 PMC1084330

[19] Miller S, Krijnse-Locker J (2008) Modification of intracellular membrane structures for virus replication. Nat Rev Microbiol 6: 363–374. 18414501 10.1038/nrmicro1890 PMC7096853

[20] Nagy PD, Pogany J (2012) The dependence of viral RNA replication on co-opted host factors. Nat Rev Microbiol 10: 137–149. 10.1038/nrmicro2692 PMC7097227 22183253

[21] Heaton NS, Randall G (2010) Dengue virus-induced autophagy regulates lipid metabolism. Cell Host Microbe 8: 422–432. 21075353 10.1016/j.chom.2010.10.006 PMC3026642

[22] Dinkins C, Arko-Mensah J, Deretic V (2010) Autophagy and HIV. Semin Cell Dev Biol 21: 712–718. 20403451 10.1016/j.semcdb.2010.04.004 PMC3108047

[23] Khakpoor A, Panyasrivanit M, Wikan N, Smith DR (2009) A role for autophagolysosomes in dengue virus 3 production in HepG2 cells. J Gen Virol 90: 1093–1103. 19264601 10.1099/vir.0.007914-0

[24] McLean JE, Wudzinska A, Datan E, Quaglino D, Zakeri Z (2012) Flavivirus NS4A-induced autophagy protects cells against death and enhances virus replication. J Biol Chem 286: 22147–22159. 10.1074/jbc.M110.192500 PMC3121359 21511946

[25] Mateo R, Nagamine CM, Spagnolo J, Mendez E, Rahe M, et al (2013) Inhibition of cellular autophagy deranges dengue virion maturation. J Virol 87: 1312–1321. 23175363 10.1128/JVI.02177-12 PMC3554187

[26] Li JK, Liang JJ, Liao CL, Lin YL (2012) Autophagy is involved in the early step of Japanese encephalitis virus infection. Microbes Infect 14: 159–168. 21946213 10.1016/j.micinf.2011.09.001

[27] Ke PY, Chen SS (2011) Activation of the unfolded protein response and autophagy after hepatitis C virus infection suppresses innate antiviral immunity in vitro. J Clin Invest 121: 37–56. 21135505 10.1172/JCI41474 PMC3007134

[28] Sir D, Kuo CF, Tian Y, Liu HM, Huang EJ, et al (2012) Replication of hepatitis C virus RNA on autophagosomal membranes. J Biol Chem 287: 18036–18043. 22496373 10.1074/jbc.M111.320085 PMC3365724

[29] Beatman E, Oyer R, Shives KD, Hedman K, Brault AC, et al (2012) West Nile virus growth is independent of autophagy activation. Virology 433: 262–272. 22939285 10.1016/j.virol.2012.08.016 PMC3444629

[30] Vandergaast R, Fredericksen BL (2012) West Nile Virus (WNV) Replication Is Independent of Autophagy in Mammalian Cells. PLoS One 7: e45800. 23029249 10.1371/journal.pone.0045800 PMC3448696

[31] Bakonyi T, Gould EA, Kolodziejek J, Weissenbock H, Nowotny N (2004) Complete genome analysis and molecular characterization of Usutu virus that emerged in Austria in 2001: comparison with the South African strain SAAR-1776 and other flaviviruses. Virology 328: 301–310. 15464850 10.1016/j.virol.2004.08.005

[32] Martin-Acebes MA, Blazquez AB, Jimenez de Oya N, Escribano-Romero E, Saiz JC (2011) West nile virus replication requires Fatty Acid synthesis but is independent on phosphatidylinositol-4-phosphate lipids. PLoS One 6: e24970. 21949814 10.1371/journal.pone.0024970 PMC3176790

[33] Martin-Acebes MA, Saiz JC (2011) A West Nile virus mutant with increased resistance to acid-induced inactivation. J Gen Virol 92: 831–840. 21228127 10.1099/vir.0.027185-0

[34] Kabeya Y, Mizushima N, Ueno T, Yamamoto A, Kirisako T, et al (2000) LC3, a mammalian homologue of yeast Apg8p, is localized in autophagosome membranes after processing. Embo J 19: 5720–5728. 11060023 10.1093/emboj/19.21.5720 PMC305793

[35] Pankiv S, Clausen TH, Lamark T, Brech A, Bruun JA, et al (2007) p62/SQSTM1 binds directly to Atg8/LC3 to facilitate degradation of ubiquitinated protein aggregates by autophagy. J Biol Chem 282: 24131–24145. 17580304 10.1074/jbc.M702824200

[36] Galindo I, Hernaez B, Munoz-Moreno R, Cuesta-Geijo MA, Dalmau-Mena I, et al (2012) The ATF6 branch of unfolded protein response and apoptosis are activated to promote African swine fever virus infection. Cell Death Dis 3: e341. 22764100 10.1038/cddis.2012.81 PMC3406580

[37] Gillespie LK, Hoenen A, Morgan G, Mackenzie JM (2010) The endoplasmic reticulum provides the membrane platform for biogenesis of the flavivirus replication complex. J Virol 84: 10438–10447. 20686019 10.1128/JVI.00986-10 PMC2950591

[38] Welsch S, Miller S, Romero-Brey I, Merz A, Bleck CK, et al (2009) Composition and three-dimensional architecture of the dengue virus replication and assembly sites. Cell Host Microbe 5: 365–375. 19380115 10.1016/j.chom.2009.03.007 PMC7103389

[39] Hariri M, Millane G, Guimond MP, Guay G, Dennis JW, et al (2000) Biogenesis of multilamellar bodies via autophagy. Mol Biol Cell 11: 255–268. 10637306 10.1091/mbc.11.1.255 PMC14772

[40] Klionsky DJ, Abeliovich H, Agostinis P, Agrawal DK, Aliev G, et al (2008) Guidelines for the use and interpretation of assays for monitoring autophagy in higher eukaryotes. Autophagy 4: 151–175. 18188003 10.4161/auto.5338 PMC2654259

[41] Klionsky DJ, Abdalla FC, Abeliovich H, Abraham RT, Acevedo-Arozena A, et al (2012) Guidelines for the use and interpretation of assays for monitoring autophagy. Autophagy 8: 445–544. 22966490 10.4161/auto.19496 PMC3404883

[42] Panyasrivanit M, Khakpoor A, Wikan N, Smith DR (2009) Co-localization of constituents of the dengue virus translation and replication machinery with amphisomes. J Gen Virol 90: 448–456. 19141455 10.1099/vir.0.005355-0

[43] Romero-Brey I, Merz A, Chiramel A, Lee JY, Chlanda P, et al (2012) Three-dimensional architecture and biogenesis of membrane structures associated with hepatitis C virus replication. PLoS Pathog 8: e1003056. 23236278 10.1371/journal.ppat.1003056 PMC3516559

[44] Bampton ET, Goemans CG, Niranjan D, Mizushima N, Tolkovsky AM (2005) The dynamics of autophagy visualized in live cells: from autophagosome formation to fusion with endo/lysosomes. Autophagy 1: 23–36. 16874023 10.4161/auto.1.1.1495

[45] Yu CY, Hsu YW, Liao CL, Lin YL (2006) Flavivirus infection activates the XBP1 pathway of the unfolded protein response to cope with endoplasmic reticulum stress. J Virol 80: 11868–11880. 16987981 10.1128/JVI.00879-06 PMC1642612

[46] Ambrose RL, Mackenzie JM (2011) West Nile virus differentially modulates the unfolded protein response to facilitate replication and immune evasion. J Virol 85: 2723–2732. 21191014 10.1128/JVI.02050-10 PMC3067947

[47] Medigeshi GR, Lancaster AM, Hirsch AJ, Briese T, Lipkin WI, et al (2007) West Nile virus infection activates the unfolded protein response, leading to CHOP induction and apoptosis. J Virol 81: 10849–10860. 17686866 10.1128/JVI.01151-07 PMC2045561

[48] Deegan S, Saveljeva S, Gorman AM, Samali A (2012) Stress-induced self-cannibalism: on the regulation of autophagy by endoplasmic reticulum stress. Cell Mol Life Sci 70: 2425–2441. 23052213 10.1007/s00018-012-1173-4 PMC11113399

[49] Klionsky DJ, Baehrecke EH, Brumell JH, Chu CT, Codogno P, et al (2011) A comprehensive glossary of autophagy-related molecules and processes (2nd edition). Autophagy 7: 1273–1294. 21997368 10.4161/auto.7.11.17661 PMC3359482

[50] Chvala S, Kolodziejek J, Nowotny N, Weissenbock H (2004) Pathology and viral distribution in fatal Usutu virus infections of birds from the 2001 and 2002 outbreaks in Austria. J Comp Pathol 131: 176–185. 15276857 10.1016/j.jcpa.2004.03.004

[51] Sir D, Chen WL, Choi J, Wakita T, Yen TS, et al (2008) Induction of incomplete autophagic response by hepatitis C virus via the unfolded protein response. Hepatology 48: 1054–1061. 18688877 10.1002/hep.22464 PMC2562598

[52] Workman P, Clarke PA, Raynaud FI, van Montfort RL (2010) Drugging the PI3 kinome: from chemical tools to drugs in the clinic. Cancer Res 70: 2146–2157. 20179189 10.1158/0008-5472.CAN-09-4355 PMC3242038

[53] Scagnolari C, Caputo B, Trombetti S, Cacciotti G, Solda A, et al (2013) Usutu Virus Growth in Human Cell Lines: Induction of and Sensitivity to Type I and Iii Interferons. J Gen Virol 94 (Pt 4) 789–95. 23255619 10.1099/vir.0.046433-0

[54] Estrabaud E, De Muynck S, Asselah T (2011) Activation of unfolded protein response and autophagy during HCV infection modulates innate immune response. J Hepatol 55: 1150–1153. 21723841 10.1016/j.jhep.2011.04.025

