# Supplementary figures and images for: Infection with Usutu Virus Induces an Autophagic Response in Mammalian Cells
Source: PLoS Negl Trop Dis. 2013 Oct 24;7(10):e2509. doi: 10.1371/journal.pntd.0002509 (PMC3812092; doi:10.1371/journal.pntd.0002509)

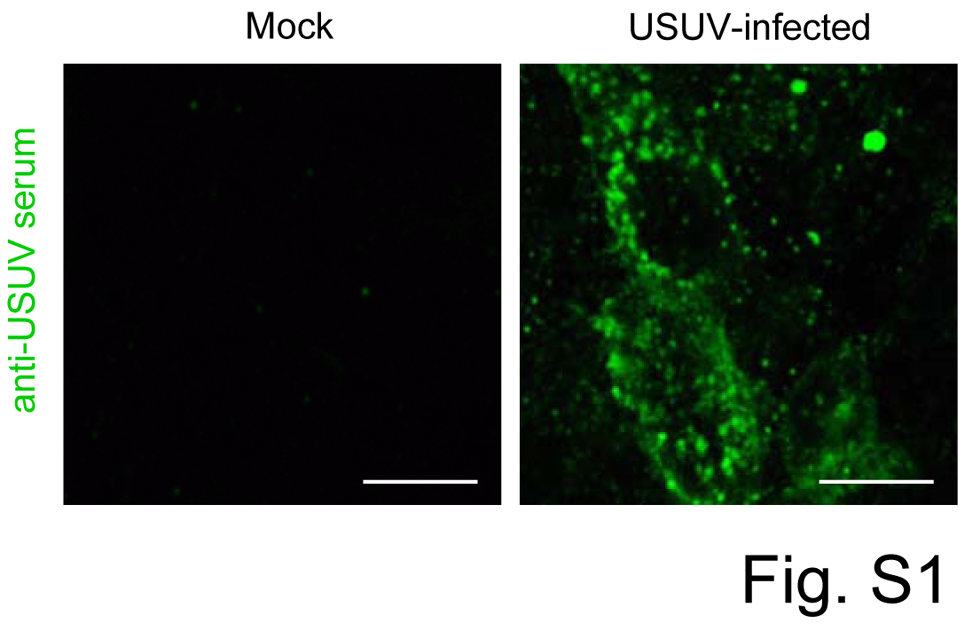

Supplement: Figure S1 — Specific staining of USUV-infected cells with a polyclonal serum. Vero cells were infected or not (mock) with USUV SAAR 1776 (MOI of 5 PFU/cell) and fixed and processed for immunofluorescence (24 h p.i.) using the serum obtained from a mouse experimentally infected with USUV. AF-488 anti-mouse IgG was used as secondary antibody. Both images were acquired using the same microscope settings. Scale bars: 10 µm. (TIF) [file pntd.0002509.s001.tif]
